# Two Cases of Venous Thromboembolism Shortly After Adenovirus-Based COVID-19 Vaccination

**DOI:** 10.7759/cureus.39609

**Published:** 2023-05-28

**Authors:** Ana Valle, Rachel Levy, Abraham Tobias, Ellen Friedman, Iman Hassan

**Affiliations:** 1 Internal Medicine, Albert Einstein College of Medicine, Bronx, USA; 2 Hematology/Oncology, Albert Einstein College of Medicine, Bronx, USA

**Keywords:** covid 19 vaccination, risk factor for vte, vaccine adverse reactions, vaccine safety, venous thromboembolism (vte)

## Abstract

As the coronavirus disease 2019 (COVID-19) global pandemic continues, multiple vaccines have been developed to decrease infection rate and number of deaths. Vaccine administration is especially important as new COVID-19 variants emerge. While the number of severe thromboembolic events reported after adenovirus-based vaccination has gained attention, there is little information regarding the presentation and management of post-vaccination venous thromboembolism (VTE). Here, we present two cases of VTE after the Janssen vaccine administration. In the first case, a 98-year-old African American female with hypertension developed bilateral lower extremity edema that evolved into unilateral lower extremity edema 20-35 days following the Janssen vaccine administration. She was found to have an extensive unilateral proximal femoral deep vein thrombosis (DVT) 35 days after the vaccination. In the second case, a 64-year-old African American female developed ecchymosis and unilateral edema six days after the Janssen vaccine administration. She was found to have proximal superficial vein thrombosis two days later. In both cases, laboratory data, including platelets and anti-heparin antibodies were within normal limits. Thus, VTE may be an adverse effect of the Janssen vaccine or any adenovirus-based vaccine, but further surveillance and investigation to elucidate this association are necessary. We advise practitioners to have a high index of suspicion for thrombosis after Janssen vaccine administration, regardless of the presence of thrombocytopenia, and avoidance of heparin products until heparin antibody results return.

## Introduction

The severe acute respiratory syndrome coronavirus 2 (SARS-CoV-2) coronavirus disease 2019 (COVID-19) global pandemic is a worldwide public health crisis with more than 674 million cases and over 6.8 million deaths [1]. COVID-19 presents with non-specific viral symptoms and shortness of breath that can progress to include the lower respiratory tract, which increases the risk of acute respiratory distress syndrome (ARDS), hypoxic respiratory failure, and death [2]. Vaccines have provided a way to curb the infection rate and the number of deaths, especially as more virulent variants develop [3,4].

To date, the United States Food and Drug Administration has granted Emergency Use Authorization for four vaccines: Johnson & Johnson/Janssen (Ad.26.COV2.S), Pfizer-BioNTech (BNT162b2), Moderna (mRNA-1273), and Novavax (Nuvaxovid and Covovax). The Johnson & Johnson/Janssen vaccine is currently recommended in the adult population as an alternative when mRNA COVID-19 vaccines are unavailable or clinically inappropriate due to the risk of vaccine-induced thrombosis with thrombocytopenia (VITT); a rare, severe thromboembolic event [5]. This phenomenon resembles heparin-induced thrombocytopenia (HIT) and is marked by venous or arterial thromboses, thrombocytopenia, and the presence of platelet-activating anti-heparin/PF4 antibodies. This phenomenon has been extensively investigated due to its catastrophic outcomes [6]. A new study suggests other types of thrombosis that present without thrombocytopenia may be more frequent following vaccination than previously thought [7].

Here, we describe two cases of VTE occurring shortly after Janssen COVID-19 vaccine administration to highlight characteristics of this complication and discuss pertinent changes in patient management when there is concern for adenovirus-vector vaccine-associated VTE. 

## Case presentation

Case 1 

**Figure 1:**
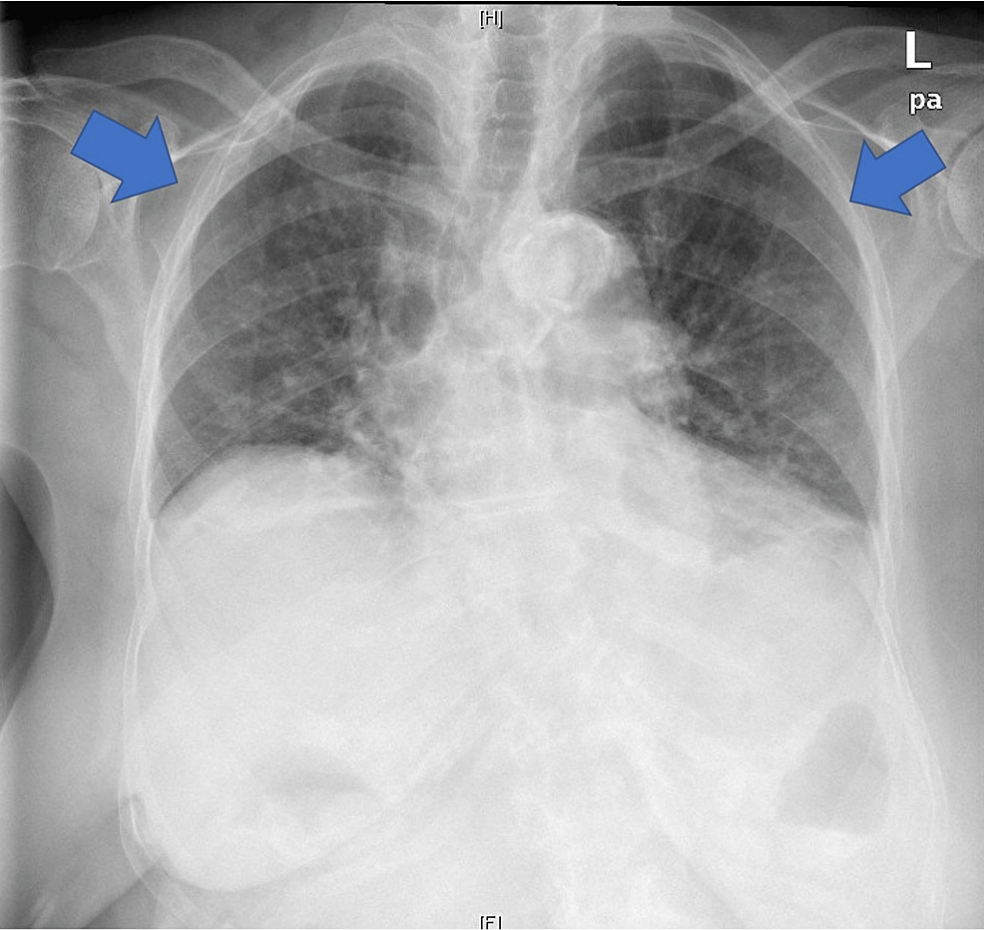
Chest x-ray showing clear lungs (arrows) without pleural effusion or pneumothorax but shallow aspiration.

A 98-year-old African American female received a single dose of the Janssen COVID-19 vaccine subcutaneously on March 16, 2021, without an immediate adverse reaction. In the days following, she endorsed body aches and mild headaches. She presented to her primary care doctor 20 days later (April 5, 2021) due to decreased exercise tolerance and was noted to have +2 pitting edema in the bilateral lower extremities. The edema was investigated with a basic metabolic panel, B-type natriuretic peptide, and chest x-ray (Figure 1).

**Figure 2:**
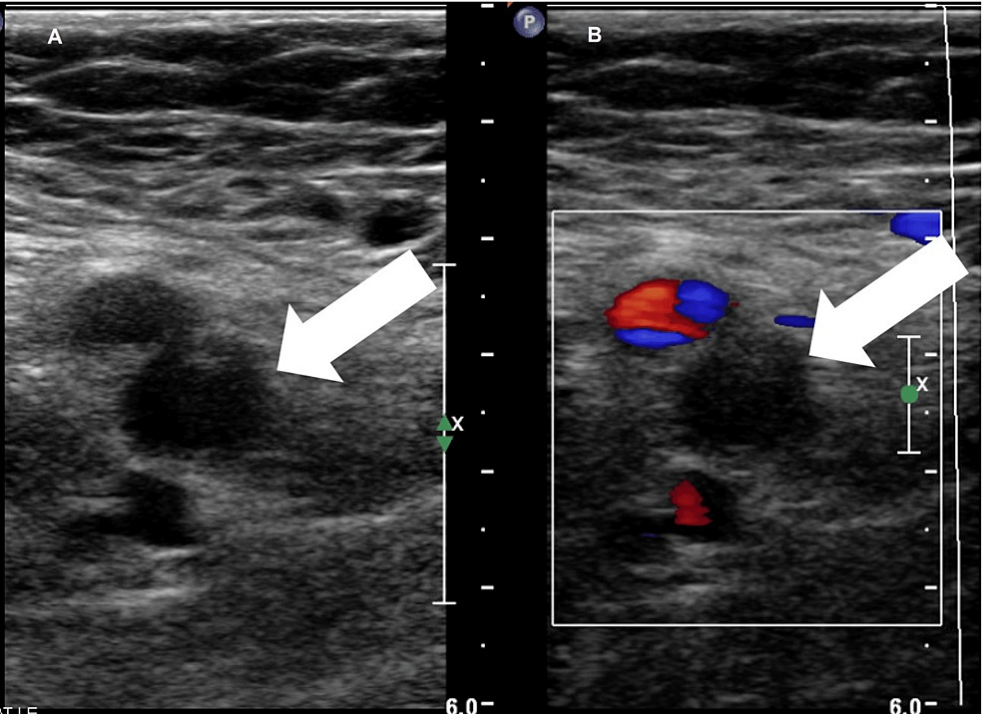
Static ultrasound image shows echogenic thrombus in the right femoral vein (Panel A). Doppler ultrasound image shows lack of bloodflow in the right femoral vein due to thrombus (Panel B)

An echocardiogram was scheduled for the upcoming month. The patient was prescribed furosemide 20 mg daily for symptomatic relief. When the aforementioned laboratory results were normal, she was sent for a lower extremity duplex ultrasound. On April 20, 2021, 35 days after vaccine administration, a duplex ultrasound revealed an acute occlusive DVT in the right proximal-mid femoral and gastrocnemius veins, along with a partially occlusive DVT of the right common and distal femoral veins (Figure 2). 

Given these findings, she was instructed to present to the emergency department for further management. On arrival, she was hemodynamically stable without fever, hypotension, or tachycardia. Her only active medical problem was hypertension. Past medical history included hepatitis C, which had been treated with sofosbuvir and simeprevir in 2015, and invasive ductal breast cancer diagnosed in 2008, which was removed with lumpectomy and treated with radiation and tamoxifen therapy completed in 2014. She follows regularly in a breast cancer survivorship program and the most recent bilateral breast ultrasound in January 2021 was negative. She denied trauma to the right lower extremity, recent travel, infection (including COVID-19), surgical procedures, or periods of prolonged immobility in the past year. She was of normal weight, has never used tobacco products, and did not have a personal nor family history of thrombotic or hemorrhagic disorders, including venous thromboembolism. The patient ambulates regularly with a walker although at times requires assistance with transfers. All her previous colonoscopies and pap smears were normal even though she no longer undergoes age-based routine malignancy screenings. A physical exam in the emergency department revealed unilateral 2+ pitting edema of the right lower extremity up to the right knee, without associated swelling, erythema, paresthesia, pain, or sensitivity to touch. Xpert® Xpress (Cepheid, Sunnyvale, California, United States) flu/respiratory syncytial virus (RSV)/SARS-CoV-2 real-time polymerase chain reaction (PCR) assay was negative for COVID-19. Complete blood count (CBC) showed a white blood count (WBC) count of 4.6 k/uL (reference range: 4.8-10.8 k/uL), hemoglobin of 12.2 g/dL (reference range: 11.2-14.7 gm/dL), hematocrit 39.6% (reference range: 36.0-45.0%), and platelet count of 235 k/uL (reference range 150-400 k/uL). A basic coagulation profile including prothrombin time (PT), partial thromboplastin time (PTT), international normalized ratio (INR), and liver function tests were within normal limits. More extensive hypercoagulable workup, including anti-heparin antibodies, anti-phospholipid antibody studies, and factor deficiencies, was initially not performed, given this was her first DVT. 

*Outcome and Follow-up* 

The patient was initially treated with one dose of weight-based therapeutic enoxaparin and then switched to apixaban on her first hospital day. She was then discharged on her second hospital day with a plan to continue standard DVT treatment of anticoagulation for three months with regular primary care follow-up. This DVT was reported on the Vaccine Adverse Event Reporting System (VAERS) as a possible adverse effect of the Janssen vaccine. 

Primary care follow-up was nine days after hospital discharge. At that time, platelets and fibrinogen were within normal limits (217 k/uL and 336 mg/dL, respectively), and anti-heparin (PF4) IgG antibodies had a normal optical density of 0.04 (reference range: 0.00-0.399 optical density). However, D-dimer was elevated to 2.17 ug/mL (reference range: 0.27-0.5 ug/mL). The patient completed three months of anticoagulation without adverse events or complications. Furthermore, the patient had no other medical incidents, which may have been adverse events secondary to vaccination, such as Bell's palsy, Guillan Barre Syndrome, cerebrovascular accidents, or myocardial infarcts. 

Case 2 

A 64-year-old African American female received a single dose of the Janssen COVID-19 vaccine subcutaneously on April 6, 2021, without an immediate adverse reaction. Six days later a family member noted a small ecchymosis on her medial left calf, which prompted the patient to present to a local emergency department on April 14, 2021. On arrival, she was hemodynamically stable without fever, hypotension, or tachycardia. Her only active medical problems were hypertension and hyperlipidemia. Past medical history included symptomatic menorrhagia due to uterine fibroids in 2005, which resolved without intervention by 2011. She denied trauma to the right lower extremity, recent travel, infection (including COVID-19), surgical procedures, or periods of prolonged immobility in the past year. Her BMI was 30 and 5 pack year history greater than 20 years prior. She ambulated independently and was up to date with her age-appropriate malignancy screenings, all of which were negative. The patient did not have a personal or family history of thrombotic or hemorrhagic disorders, including venous thromboembolism. A physical exam in the emergency department revealed unilateral 2+ pitting edema of the right lower extremity below the right knee and a small ecchymosis on her medial left calf without associated erythema, paresthesia, pain, or sensitivity to touch. The flu/SARS-CoV-2 real-time PCR assay was negative for COVID-19. CBC showed a WBC count of 6.9 k/uL (reference range: 4.5-11 k/uL), hemoglobin of 13 gm/dL (reference range: 11.2-14.7 gm/dL), hematocrit 39.8% (reference range: 36.0-45.0%), and platelet count of 276 k/uL (reference range 150-400 k/uL). A basic coagulation profile revealed a mildly elevated PT of 13.8 seconds (reference range: 9.4-13.5) with INR of 1.1 (reference range: 0.8-1.1) and PTT of 33.5 seconds (reference range: 24.7-36.3 seconds). Liver function tests were within normal limits except for a mild elevation in alkaline phosphatase of 137 (reference range: 38-126 U/L). D-dimer was elevated to 772 ng/mL (DDU) (reference range: 0-243 ng/mL (DDU)) and anti-heparin (PF4) IgG antibodies had a normal optical density of 0.107 (reference range: 0.00 - 0.4 optical density). More extensive hypercoagulable workup, anti-phospholipid antibody studies, and factor deficiencies were initially not performed given this was her first DVT. Left lower extremity duplex ultrasound revealed a non-occlusive superficial thrombosis in the greater saphenous vein at the level of the mid-calf (image unavailable). 

Outcome and Follow-Up 

The patient received one dose of apixaban and shortly afterward was unable to speak due to a locked jaw and back arching. She was transitioned to warfarin and developed similar symptoms six days later. Neurology evaluated the patient and deemed the presentation most consistent with psychogenic non-epileptiform seizures given CT head and electroencephalogram were normal. Warfarin was discontinued and replaced with fondaparinux, which the patient continued without incidence or adverse effects until March 25, 2022, when her outpatient hematologist repeated a lower extremity duplex, which was negative for any deep or superficial venous thrombosis. During this period the patient had no other documented medical incidents which may have been adverse events secondary to vaccination, such as Bell's palsy, Guillan Barre Syndrome, cerebrovascular accidents, or myocardial infarcts. 

## Discussion

Here we present the cases of two African American females aged over 60 years, who were found to have VTE within a month of receiving a single-dose adenovirus COVID-19 vaccine (Table 1). 

**Table 1:** Comparison of history, presentation, treatment, and outcomes of Case 1 and Case 2 PT: prothrombin time; ALP: alkaline phosphotase; VTE: venous thromboembolism; DVT: deep vein thrombosis; COVID-19: coronavirus disease 2019; PCR: polymerase chain reaction

Patient Variables	Case 1	Case 2
Age	98	64
Sex	Female	Female
Race	Black	Black
Past Medical History	Treated Hepatitis C	Hypertension
	Breast cancer (in remission)	Hyperlipidemia
Initial Presentation	Decreased exercise tolerance and bilateral lower extremity edema	A small, painless ecchymosis on the medial aspect of the left calf
VTE Location	Right proximal-mid femoral and gastrocnemius veins	Non-occlusive superficial thrombosis in left greater saphenous vein at level of mid-calf
	Partially occlusive DVT of right common and distal femoral veins	
Laboratory results		
Leukocytes	Mild leukopenia	Normal
Hemoglobin	Normal	Normal
Platelets	Normal	Normal
Coagulation profile	Normal	Mildly elevated PT
Liver tests	Normal	Mildly elevated ALP
COVID-19 PCR	Negative	Negative
D-dimer	Elevated	Elevated
Fibrinogen	Normal	N/A
PF4 IgG antibodies	Normal	Normal
Management	One dose of therapeutic enoxaparin followed by apixaban for 3 months	One dose of apixaban followed by six doses of warfarin followed by fondaparinux
VTE Complications	None	None
Other Vaccine Adverse Events	None	None

**Figure 3:**
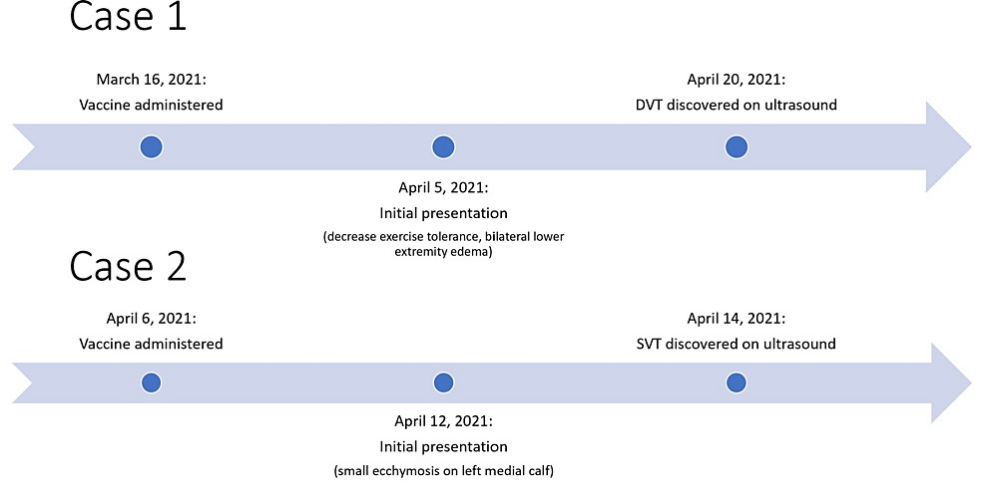
Timeline of vaccine administration, patient presentation, and VTE detection in Case 1 and Case 2. VTE: venous thromboembolism

In Case 1, the patient developed symptoms 20 days after vaccine administration, and DVT was confirmed 35 days after vaccine administration. In Case 2, the patient developed symptoms six days after vaccine administration and SVT was discovered eight days after vaccination. These timelines can be seen in Figure 3. This timing begs the question of whether the vaccine administered may have contributed to these cases of VTE. 

In Europe, there have been multiple reports that both adenovirus-based ChAdOx1 nCoV-19 (Vaxzeria, formerly known as AstraZeneca) and Ad26.COV2. S have been associated with VITT, which has not been reported as extensively after vaccination with mRNA vaccines that do not contain an adenovirus vector [6,8]. It has been hypothesized that the adenovirus vector may be a trigger for micro-trauma and these findings have been replicated in animal models [6,9]. It is also possible that Spike protein variants inadvertently bind to endothelial cells, which drives an immune response [9]. While neither of these patients had thrombocytopenia or heparin antibodies, micro-trauma or cell activation from the Ad26.COV2. S vaccine may have caused endothelial damage or dysfunction to trigger an immune response within the venous system, which led to thrombosis.

Endothelial damage is a well-reported mechanism in the pathophysiology of VTE formation. Along with hypercoagulability and venous stasis, it forms Virchow’s triad [10]. Although the patient in Case 1 has a history of a hypercoagulable state, she regularly underwent surveillance to ensure she did not have a recurrence of breast cancer. Additionally, she ambulates often and did not have any recent procedures, which makes immobility leading to venous stasis less likely. Similarly, the second patient’s history of menorrhagia resolved a decade prior to vaccine administration. Although elderly age, hypertension, and increase in BMI have been associated with increased DVT risk, both patients had a baseline low Wells score for DVT [11]. Recently Cari et al. found that the adenovirus-based COVID-19 vaccines ChAdOx1 nCoV-19 and Ad26.COV2.S have comparable risk of thromboembolic disease to each other and these frequencies were higher than expected [7]. While these findings have yet to be replicated, they support our suspicion that the VTEs reported here were connected to the Janssen vaccine. 

If Janssen vaccine administration is not considered a provoking factor, these VTE would be considered unprovoked. In the years prior to COVID-19, the estimated incidence rate of a DVT in a female aged >84 was 1206 per 100,000 person-years and the incidence of SVT is thought to be even more frequent [12,13]. Furthermore, it is known that VTE, particularly DVT, are more frequent in women of all ages in comparison to their male peers [12]. Thus, these post-vaccination VTEs may in fact be expected based on the age and sex of these patients. 

While the clinical scenarios described above are not as unique and severe as VITT, it is still important to investigate all thrombotic events following COVID-19 vaccine administration in order to understand the possible vaccine side effects, inform healthcare providers of those complications, and provide anticipatory guidance to patients. In fact, it has been shown that the majority of VTE events after adenovirus-based COVID-19 vaccine administration were not associated with thrombocytopenia [11]. Thus, while thrombocytopenia may be a factor for a more severe prognosis, it does not exclude the presence of thrombosis. Furthermore, heparin products could have been avoided in Case 1 until anti-heparin/PF4 antibody results were obtained or the possibility of VITT was ruled out. Currently, Janssen is considered an alternative to the mRNA COVID-19 vaccines when the latter may be clinically inappropriate or if a patient would only receive COVID-19 vaccination with Janssen based on its risk of VITT and Guillain-Barré syndrome [14]. This emphasizes that while there are risks with the Janssen vaccine, it may still be clinically appropriate for specific populations. 

## Conclusions

Limited information is available regarding the risk of VTE after COVID-19 vaccination. Here we present two cases of VTE in the month following the Janssen vaccine administration in individuals with relatively low VTE risk. Based on these cases and new data, it is possible that VTE may be an adverse effect of the Janssen vaccine or any adenovirus-based vaccine but further surveillance and investigation to elucidate this association is necessary. This information is crucial to provide anticipatory guidance to patients. Until we gain further knowledge of VTE risk after adenovirus-based vaccine administration, we suggest an early ultrasound duplex to evaluate for VTE and avoiding heparin products in patients with clinical symptoms concerning for VTE following adenovirus-based COVID-19 vaccination. 
